# Development and validation of a nomogram for predicting intellectual disability in children with cerebral palsy

**DOI:** 10.1016/j.ijchp.2024.100493

**Published:** 2024-08-31

**Authors:** Junying Yuan, Gailing Wang, Mengyue Li, Lingling Zhang, Longyuan He, Yiran Xu, Dengna Zhu, Zhen Yang, Wending Xin, Erliang Sun, Wei Zhang, Li Li, Xiaoli Zhang, Changlian Zhu

**Affiliations:** a Henan Pediatric Clinical Research Center and Henan Key Laboratory of Child Brain Injury, Institute of Neuroscience and Third Affiliated Hospital and of Zhengzhou University, Zhengzhou 450052, China; b Cerebral Palsy Rehabilitation Center, Third Affiliated Hospital of Zhengzhou University, Zhengzhou 450052, China; c Center for Child Behavioral Development, The Third Affiliated Hospital of Zhengzhou University, Zhengzhou 450052, China; d Center for Brain Repair and Rehabilitation, Institute of Neuroscience and Physiology, University of Gothenburg, Gothenburg 40530, Sweden

**Keywords:** Cerebral palsy, Intellectual disability, Prediction model, MRICS, GMFCS, Epilepsy

## Abstract

**Objective:**

Intellectual disability (ID) is a prevalent comorbidity in children with cerebral palsy (CP), presenting significant challenges to individuals, families and society. This study aims to develop a predictive model to assess the risk of ID in children with CP.

**Methods:**

We analyzed data from 885 children diagnosed with CP, among whom 377 had ID. Using least absolute shrinkage and selection operator regression, along with univariate and multivariate logistic regression, we identified key predictors for ID. Model performance was evaluated through receiver operating characteristic curves, calibration plots, and decision curve analysis (DCA). Bootstrapping validation was also employed.

**Results:**

The predictive nomogram included variables such as preterm birth, CP subtypes, Gross Motor Function Classification System level, MRI classification category, epilepsy status and hearing loss. The model demonstrated strong discrimination with an area under the receiver operating characteristic curve (AUC) of 0.781 (95% CI: 0.7504-0.8116) and a bootstrapped AUC of 0.7624 (95% CI: 0.7216-0.8032). Calibration plots and the Hosmer-Lemeshow test indicated a good fit (χ^2^= 7.9061, p = 0.4427). DCA confirmed the model's clinical utility. The cases were randomly divided into test group and validation group at a 7:3 ratio, demonstrating strong discrimination, good fit and clinical utility; similar results were found when stratified by sex.

**Conclusions:**

This predictive model effectively identifies children with CP at a high risk for ID, facilitating early intervention strategies. Stratified risk categories provide precise guidance for clinical management, aiming to optimize outcomes for children with CP by leveraging neuroplasticity during early childhood.

## Introduction

Cerebral palsy (CP) is defined as a movement and posture disorder that results in activity limitation due to no progressive disturbances in the developing brain, often accompanied by various impairments (Rosenbaum et al., 2007). Among these, intellectual disability (ID) is particularly prevalent, though there is significant variability in estimates regarding its prevalence in children with CP. Western countries with national registries report prevalence rates of about 30% to 42.01% (Bufteac Gincota, Jahnsen, Spinei, & Andersen, 2021; Cummins et al., 2021; Himmelmann et al., 2006, Sigurdardottir et al., 2008). A population-based study based on the Victorian CP Register reported a prevalence of 45% (Reid et al., 2018). A meta-analysis in 2023 found the prevalence to be 37.2% (95%CI 26.7-48.3%) (Sattoe & Hilberink, 2023). Additionally, a systematic review reported that half of the children with CP have ID (Novak et al., 2012). Another recent meta-analysis of studies from China reported the prevalence of 58.0% (95% CI: 51.8-64.3%) (Gong et al., 2023). ID significantly impacts daily activities, caregiver burden, quality of life, and longevity (Aguayo et al., 2019; Olusanya et al., 2022). Furthermore, motor and intellectual abilities are interdependent, especially during early developmental stages (Casey et al., 2005). However, ID can typically only be diagnosed in children older than 4 or 5 years of age using IQ tests and assessments of adaptive capacity, or through clinical features in those unable to complete such assessment. Delays in diagnosis of ID may hinder timely interventions that leverage early neuroplasticity to improve outcomes (Yuan et al., 2018).

ID is challenging to predict because it encompasses a heterogeneous group of conditions that affect intellectual functioning and adaptive behaviors. A study from the health database of the Nice Region developed a diagnostic model to identify severe ID in teenagers with CP (Bertoncelli et al., 2019). This model was based on data from 21 subjects with severe ID out of 486 individuals with CP. Although the development of the predictive learning model was detailed, its validation and calibration were not provided (Bertoncelli et al., 2019). Additionally, focusing on teenagers may miss the optimal time for intervention, and the study's small sample size (only 21 subjects with severe ID) could limit the reliability of the findings (Bertoncelli et al., 2019).

To explore the comorbidities associated with ID in CP, we utilized a case-control design within a large CP cohort to develop and validate a logistic regression diagnostic model. This model aims to identify risk factors associated with ID in CP. This study adheres to the Transparent Reporting of a multivariable prediction model for Individual Prognosis or Diagnosis guidelines or multivariable prediction model research (Collins et al., 2015; Wolff et al., 2019). Additionally, to minimize the risk of bias, the Prediction model Risk of Bias Assessment Tool was employed as a guiding checklist for this study (Wolff et al., 2019) .

## Materials and methods

### Study population

Data were collected from pediatric patients diagnosed with CP who underwent rehabilitation interventions at the Child Rehabilitation Center of the Third Affiliated Hospital between January 1, 2011 and December 31, 2020. Pediatric neurologists or child rehabilitation doctors conducted comprehensive evaluations, which included assessing maternal, perinatal, and postnatal risk factors, developmental milestones, medical history, neurological examinations, routine cerebral imaging, and laboratory tests. Metabolic or genetic analyses were recommended for children with normal brain imaging results and no evident high-risk factors. Children whose last admission to the center was less than 24 months ago were followed up until they reached at least 24 months of age. Those found to have abnormal genetic or metabolic features explaining the clinical features of "CP" were excluded from the CP group during hospitalization or follow-up (Yuan et al., 2024). Only cases with comprehensive data were included in the analysis.

### Sample size calculation and predictor selection

The sample size was calculated using the methodology proposed by Riley and implemented through the pmsampsize package (Riley et al., 2020). With an observed ID rate of 43% among children with CP and a c-index (Bertoncelli et al., 2019) of 0.74, the necessary sample size was calculated to be 836. This sample size is adequate to develop and validate a clinical prediction model effectively.

Predictor variables were chosen based on various factors associated with CP, categorized into maternal, perinatal, and postnatal risk factors (Sadowska et al., 2020; Yuan et al., 2019). Maternal factors included advanced maternal age, adverse pregnancy histories (Qian et al., 2023), family address, and accompany diseases during pregnancy. Perinatal risk factors such as perinatal adversity, gestational age, sex, multi-birth, intrauterine growth retardation, gravidity, and parity were also considered. Additionally, postnatal risk factors, clinical features and comorbidities, such as Gross Motor Function Classification System (GMFCS) (Dalvand et al., 2012), MRI classification system (MRICS) (Reid et al., 2018), CP subtypes (Noten et al., 2022), epilepsy (Reid et al., 2018), hearing loss (Reid et al., 2018), and visual impairments (Reid et al., 2018) were included. The selection of these predictors was informed by previous studies indicating their association with ID in individuals with CP (Bertoncelli et al., 2019; Cummins et al., 2021; Huang et al., 2016; Reid et al., 2018).

### Data management and definition

ID was identified in children aged 4 years and older, through the use of IQ tests with scores below 70 and evaluations of limited adaptive functioning. In cases where children were unable to complete standard intelligence assessments due to their condition, ID was determined through clinical observations, with only these estimations being deemed reliable (Stadskleiv, 2020). Maternal age was dichotomized into advanced maternal age (≥35 years) and no-advanced maternal age (younger than 35 years) (Attali & Yogev, 2021; Schneider et al., 2018). Adverse pregnancy histories included spontaneous abortions and stillbirths. Maternal conditions included in the analysis were hypertension, diabetes, pre-eclampsia and eclampsia. Preterm birth was defined as delivery before 37 weeks of gestation. Birth weight was classified as low birth weight (<2500 g) and normal birth weight (≥2500 g) (Başaran et al., 2023). Intrauterine growth retardation was characterized by fetal weight falling below the 10th percentile for gestational age (Vandenbosche & Kirchner, 1998), as determined by the standards derived from a study encompassing 28 provinces in China (Li et al., 2015). Definitions for CP subtypes, epilepsy, learning loss, visual impairment, GMFCS, MRICS were established prior to the initiation of this study (Yuan et al., 2024).

### Statistical analysis

The data were presented as counts and percentages and analyzed using R (V 4.3.2; https://www.R-project.org) and Stata 17. Univariate analysis was performed using Tableone package to identify significant variables (p<0.1). The LASSO method (glmnet package) was utilized to avoid collinearity and identify optimal predictors for the ID risks in children with CP. Predictors with a significance level of *p* < 0.1 in univariate analysis were considered for LASSO analysis, and predictors with nonzero coefficient in LASSO analysis (logλ1_SE) were used to establish the nomogram of clinical prediction model (Hmisc package; rms package). Statistical tests were conducted as two-sided, ensuring a comprehensive evaluation of the results.

The prediction model was validated across three aspects (Iasonos et al., 2008): 1) Discrimination: The model's discrimination ability was evaluated using receiver operating characteristic (ROC) curves, and the area under the ROC curve (AUC). An AUC value greater than 0.7 was deemed to be indicative of robust predictive performance. Furthermore, a corrected C-index was calculated using bootstrapping with 1000 replicates to further confirm the model's discriminative capacity. 2) Calibration: The calibration of the model was assessed through calibration plots (rms packages) and the Hosmer-Lemeshow test (ResourceSelection package) (Lele & Keim, 2006). A Hosmer - Lemeshow test with a *p* value > 0.05 indicated satisfactory calibration, validating that the predicted probabilities closely matched the actual outcomes. 3) Clinical Utility: The clinical utility of the model was evaluated using decision curve analysis (DCA) (rmda package), which measured the net benefits across various threshold probabilities in the entire cohort to assess its clinical practicality.

## Results

### Study cohort

A total of 885 children aged 48 months and older (range: 48 -189 months) with CP were included in the study, all of whom had complete datasets. Within the sample population, 42.60% (377 out of 885 were identified as having ID, with 78.25% (295 out of 377) categorized as mild ID, and 21.75% (82 out of 377) as severe ID. Additionally, 69.88% (618 out of 885) of these children were male, with an average age of 80.11 ± 26.32 months. The 885 CP children were randomly classified into the derivation (n = 599) and validation sets (n = 256) at a 7:3 ratio. Further details regarding the demographic characteristics of the children can be found in Table 1.

**Table 1:** Clinical and demographic characteristics of the cohort.

	Without ID (n = 508)	With ID (n = 377)	p
**Maternal, prenatal and gestational risk factors**			
Advanced maternal age, N (%)	108 (21.3)	100 (26.5)	0.081
Urban, N (%)	112 (22.0)	70 (18.6)	0.237
Adverse pregnancy history, N (%)	19 (3.7)	18 (4.8)	0.555
Accompany diseases during pregnancy, N (%)	78 (15.4)	57 (15.1)	0.999
**Perinatal risk factors**			
Perinatal adversity, N (%)	199 (39.2)	157 (41.6)	0.502
Twins or multibirth, N (%)	49 (9.6)	24 (6.4)	0.103
Intrauterine growth retardation, N (%)	90 (17.7)	64 (17.0)	0.843
Gravidity ≥3, N (%)	92 (18.1)	80 (21.2)	0.285
Parity ≥3, N (%)	55 (10.8)	55 (14.6)	0.115
Cesarean delivery, N (%)	233 (45.9)	173 (45.9)	>0.999
Female, N (%)	148 (29.1)	119 (31.6)	0.481
Birth weight <2500g, N (%)	220 (43.3)	124 (32.9)	0.002
Preterm, N (%)	252 (49.6)	127 (33.7)	<0.001
**Clinical features and comorbidities**			
Gross motor function classification system, N (%)			<0.001
1-II	417 (82.1)	175 (46.4)	
III	59 (11.6)	72 (19.1)	
IV-V	32 (6.3)	130 (34.5)	
CP subtypes, N (%)			<0.001
Hemiplegia	163 (32.1)	39 (10.3)	
Diplegia	270 (53.1)	173 (45.9)	
Quadriplegia	48 (9.4)	106 (28.1)	
Mixed	8 (1.6)	30 (8.0)	
Dyskinesia	15 (3.0)	24 (6.4)	
Ataxia	4 (0.8)	5 (1.3)	
MRI classification system, N (%)			<0.001
Predominant white matter injury	284 (55.9)	141 (37.4)	
Maldevelopment	21 (4.1)	40 (10.6)	
Predominant grey matter injury	64 (12.6)	67 (17.8)	
Miscellaneous	79 (15.6)	68 (18.0)	
Normal	60 (11.8)	61 (16.2)	
Epilepsy, N (%)	46 (9.1)	75 (19.9)	<0.001
Hearing loss, N (%)	24 (4.7)	54 (14.3)	<0.001
Visual impairment, N (%)	40 (7.9)	45 (11.9)	0.056

### Variable selection

**Fig. 1:**
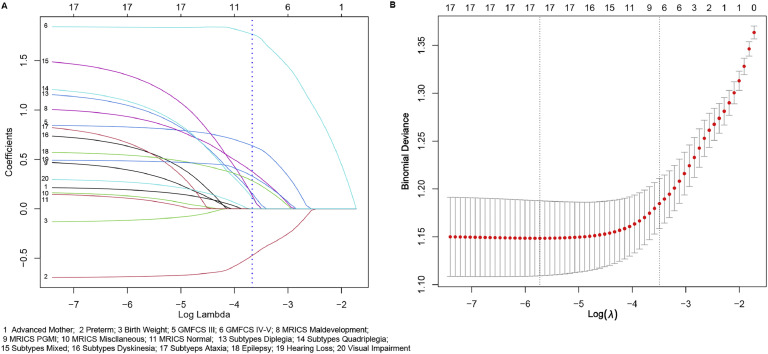
**Selection of demographic and clinical features using the lasso regression model. A**. The coefficients of the variables are plotted against log (lambda). A blue dotted vertical line indicates one standard error of the lambda from ten-fold cross-validation, marking the optimal lambda. This resulted in the selection of 8 features with nonzero coefficients. **B**. The LASSO regression model was employed with ten-fold cross-validation based on the minimum criteria. The plot illustrates log (lambda) on the x-axis and binomial deviance on the y-axis. The dotted vertical lines represent the minimum lambda (0.003263) and one standard error of the lambda (0.03043) on the log (lambda) scale.

In this study, 9 variables that achieved a *p* < 0.1 were subsequently analyzed using LASSO logistic regression, of which 8 demonstrated nonzero coefficients with a penalty parameter (λ) (Fig 1). Variables with p < 0.05 including preterm birth, GMFCS levels, MRICS, CP subtypes, and epilepsy were identified as independent predictors for ID development on multivariate logistic regression analysis. Although the variable hearing loss had a p-value of 0.0848, considering the increased risk of hearing problems in people with ID (Willems et al., 2022) and its OR (1.6767), we included hearing loss in our prediction model. Based on these results, a new predictive equation was established: Logit P = −1.2798 - 0.7685*(preterm) + 0.8278*(GMFCS III-V) + 1.8527*(GMFCS, V) + 1.2072*(Subtypes, diplegia) + 1.2404*(Subtypes, quadriplegia) + 1.5496*(Subtypes, mixed) + 0.815* (Subtypes, dyskinesia) + 0.8859 *(Subtypes, ataxia) +1.0343*(MRICS, maldevelopment) + 0.4974*(MRICS, PGMI) + 0.1878*(MRICS, miscellaneous) + 0.1941*(MRICS, normal) + 0.6165*(epilepsy) + 0.5168*(MRICS, hearing loss). The details of the prediction equation are shown in Table 2, which includes the odds ratios (ORs) indicating the strength of each independent variable's contribution to the outcome. Table 2 also provides the estimated beta coefficient for the independent variables, where β = ln (OR). The results of multivariate logistic regression analysis show that the ORs and β coefficients are adjusted or weighted (Stoltzfus, 2011).

**Table 2:** Multivariate logistic regression analysis of independent risk factors.

	β	SE	z value	p value		OR		95% CI	
Preterm, Yes	-0.7685	0.1891	-4.064	<0.001	0.4637		[0.3192, 0.6703]		
GMFCS III	0.8278	0.2116	3.913	0.0001	2.2883		[1.5132, 3.4723]		
GMFCS IV-V	1.8527	0.3135	5.909	<0.001	6.377		[3.4831, 11.9389]		
Subtypes Diplegia	1.2073	0.2368	5.098	<0.001	3.3444		[2.1222, 5.3787]		
Subtypes Quadriplegia	1.2404	0.3405	3.643	0.0003	3.457		[1.7739, 6.7593]		
Subtypes Mixed	1.5496	0.5194	2.983	0.0029	4.7096		[1.7415, 13.5603]		
Subtypes Dyskinesia	0.815	0.443	1.84	0.0658	2.2592		[0.9487, 5.4288]		
Subtypes Ataxia	0.8859	0.7592	1.167	0.2432	2.4252		[0.5407, 11.3721]		
MRICS Maldevelopment	1.0343	0.339	3.051	0.0023	2.8131		[1.4566, 5.5276]		
MRICS PGMI	0.4974	0.2722	1.828	0.0676	1.6444		[0.9648, 2.8094]		
MRICS Miscellaneous	0.1878	0.2332	0.805	0.4208	1.2066		[0.762, 1.9033]		
MRICS Normal	0.1941	0.2532	0.766	0.4434	1.2142		[0.7376, 1.993]		
Epilepsy, Yes	0.6165	0.2343	2.631	0.0085	1.8524		[1.1721, 2.9418]		
Hearing loss, Yes	0.5168	0.2998	1.724	0.0848	1.6767		[0.9378, 3.0512]		
Intercept	-1.7298	0.2385	-7.253	<0.001	0.1773		[0.1094, 0.2792]		

### Fitting the model

**Fig. 2:**
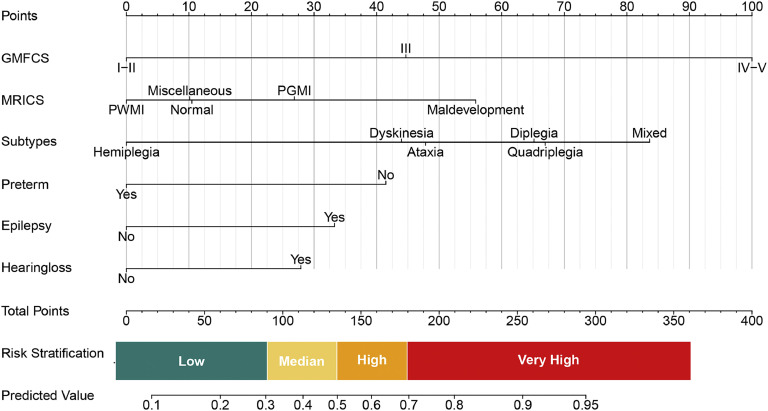
**Predictive nomogram for ID in Children with CP.** The predictive nomogram was developed using the following variables: GMFCS, MRICS, CP subtypes, preterm birth, epilepsy, and hearing loss. It translates predicted probabilities into points on a scale from 0 to 100, displayed above the figure. The total points accumulated from all predictors correspond to the predicted probability of ID in children with CP.

The model developed through univariable analysis and lasso regression was applied to the study participants and is depicted in a nomogram plot in Fig. 2. Each predictor's point can be determined by drawing a vertical line to the corresponding axis, and the total points are calculated by summing the points of each relevant factor in the nomogram. The model exhibited a sensitivity of 81.43% (95% CI: 77.45%-85.41%) and a specificity of 61.02% (95% CI: 56.5%-65.16%) at the threshold defined by the Youden index. Moreover, this high value of the Youden's index demonstrates the positive predictive value was 60.79%, and the negative predictive value was 81.58%.

### Model interpretation

The interpretation of the prognostic model is illustrated in Fig. 2, indicating that approximately 38.77% of children with CP are projected to be classified as low-risk, with a probability of less than 0.3. This subgroup exhibits a relative risk (RR) of 0.43 comparisons to the baseline population prevalence of 45% (Table 1 and Supplementary Table 1). Additional RR values for various predictive factors are provided in Supplementary Table 1.

Based on these calculated RRs, risk thresholds were stratified within the risk model as illustrated in Fig. 2. Various strategies are suggested based on different risk levels: individuals with predictive values ranging from 0 to 0.3 are advised to undergo follow-up concentrating on cognition and comprehensive language; those with values between 0.3 and 0.5 are recommended to receive outpatient intervention and follow-up; and individuals with values exceeding 0.5 are strongly encouraged to undergo inpatient intervention.

### Model performance

**Fig. 3:**
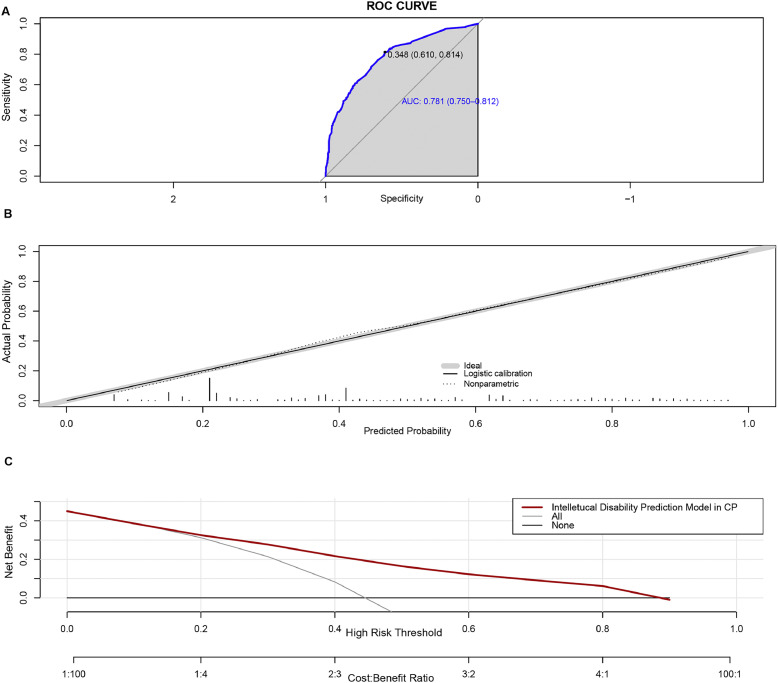
**Performance of the predictive nomogram. A**. The x-axis represents specificity, and the y-axis represents sensitivity of the model. The shaded area denotes the area under the ROC curve. **B**. The x-axis shows the predicted risk of ID, while the y-axis shows the actual probability. The dotted line represents perfect prediction by an ideal model, and the solid line indicates the performance of the nomogram. **C.** Decision curve analysis. The y-axis measures the net benefit. The red line represents the risk association with the model. The label “All” indicates the assumption that all children with CP have an ID. The “None” line represents the assumption that no children with CP have ID.

The AUC value of the prediction model was 0.781 (95% CI: 0.7504-0.8116). Bootstrapping validation with 1000 repetitions confirmed the AUC value to be 0.7624 (95% CI: 0.7216 - 0.8032). The Hosmer–Lemeshow test was not significant (χ^2^= 7.9061, *p* = 0.4427), indicating satisfactory calibration of the model. The ROC plot and calibration curve are shown in Fig. 3A and 3B. The mean probability of the cohort without ID was 0.3231 ± 0.1877, while the cohort with ID had a mean probability of 0.5648 ± 0.2392, demonstrating a significant difference (t = -16.248, *p* < 0.001).

DCA was employed to assess the clinical efficacy of the predictive nomogram. The decision curve depicted in Fig. 3C demonstrates that within a threshold probability range of 0.15 - 0.9, employing the model for predicting the likelihood of ID in children with CP would yield a favorable net benefit.

**Fig. 4:**
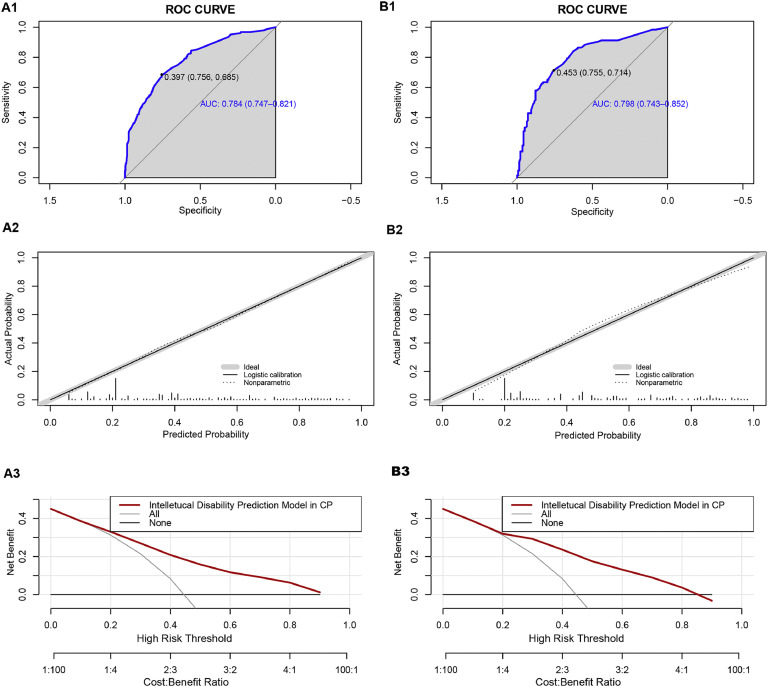
**Performance of the prediction model in the test and validation group. A1** ROC curve of the multivariate prediction model in the test group; **A2** Calibration curve of the multivariate prediction model in the test group; **A3** DCA of the model in the test group. **B1** ROC curve of the multivariate prediction model in the validation group; **B2** Calibration curve of the multivariate prediction model in the validation group; **B3** DCA of the model in the validation group.

We classified all the cases into test and validation groups at a 7:3 ratio, respectively (set random seed: 20240608). The results of AUC value, calibration plot and DCA analysis from the validation group were similar to those from the test group. In the test group, the goodness-of-fit χ2 of ID in CP children was 4.8849 (*p* = 0.7698), and in validation group, it was 8.9001(p = 0.2599), indicating no evidence of poor fit between observation and prediction (Fig. 4A2 and Fig. 4B2). Additionally, the ROC curve revealed an AUC value of 0.784 (95% CI: 0.7474-0.8206) in test group and 0.7975 (95% CI: 0.7433-0.8518) in validation group (Fig. 4A1 and Fig. 4A2). Moreover, the prediction model demonstrated a high net benefit in predicting ID probability among CP children by DCA analysis in both the test and validation groups (Fig. 4C1 and Fig. 4C2).

**Fig. 5:**
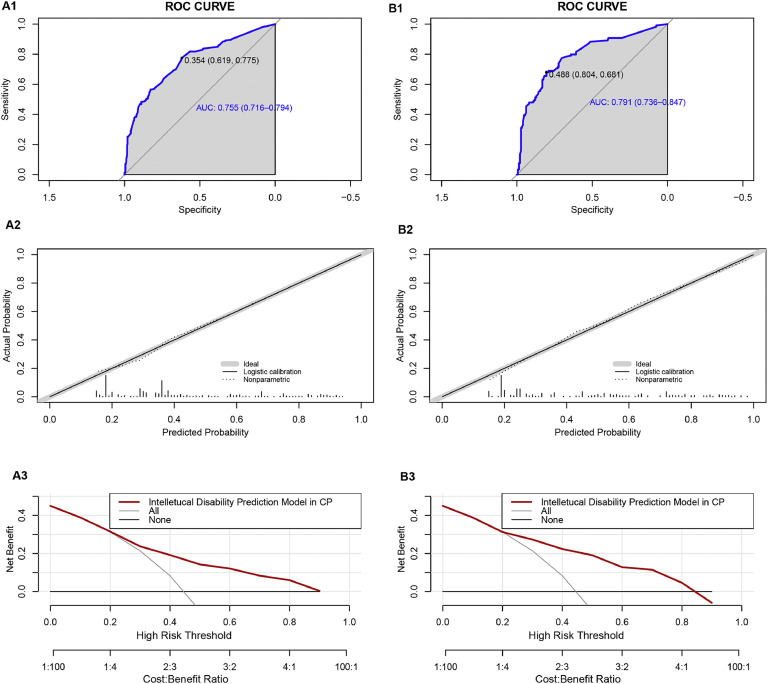
**Performance of the prediction model in the male and female groups. A1** ROC curve of the multivariate prediction model in the male group; **A2** Calibration curve of the multivariate prediction model in the male group; **A3** DCA of the model in the male group. **B1** ROC curve of the multivariate prediction model in the female group; **B2** Calibration curve of the multivariate prediction model in the female group; **B3** DCA of the model in the female group.

There were 618 male children and 267 female children in our cohort. In the male group, the goodness-of-fit χ2 of the prediction model was 7.8801 (*p* = 0.3433), and in the female group, it was 8.4207 (*p* = 0.297), indicating no evidence of poor fit between observation and prediction (Fig. 5A2 and Fig. 5B2). Additionally, the ROC curve revealed an AUC value of 0.7548 (95% CI: 0.7156-0.7941) in the male group and 0.7912 (95% CI: 0.7357-0.8468) in the female group (Fig. 5A1 and Fig. 5B1). Furthermore, the prediction model showed a high net benefit in predicting ID probability among CP children by DCA analysis in both the male and female groups (Fig. 5A3 and Fig. 5B3).

## Discussion

The nomogram was developed using several predictors identified through rigorous statistical methods, including preterm birth, CP subtypes, GMFCS levels, MRICS, and epilepsy. The model showed strong discrimination with an AUC of 0.781 and robust calibration, indicating it accurately predicts the likelihood of ID in children with CP. The results demonstrate the effectiveness of the nomogram in predicting ID, emphasizing its clinical utility for early intervention.

This predictive model is significant for clinicians as it allows for the early identification of children at high risk for ID. Such early identification is crucial because it enables timely interventions, leveraging the neuroplasticity in young children to improve outcomes. The model stratifies risk into four categories: low (<0.3), medium (0.3-0.5), high (0.5-0.7), and very high (>0.7), providing clear guidelines for clinical management. For example, children in the low-risk category may benefit from regular follow-ups focusing on cognitive and language development, while those in higher-risk categories may require more intensive interventions, including inpatient care (Aguayo et al., 2019; Olusanya et al., 2022).

Among the predictors, preterm birth was identified as a protective factor (OR = 0.4637, 95% CI: 0.3192-0.6703), which is counterintuitive given its general association with developmental issues. This finding aligns with other studies (Cummins et al., 2021; Dolk et al., 2006; Hemming et al., 2008; Reid et al., 2018) suggesting that term births might have underlying genetic or metabolic factors leading to CP, while preterm CP is often due to hypoxia which predominantly affects motor functions more than intellectual abilities (Back, 2017; Lear et al., 2022). Additionally, CP occurring in the late third trimester is primarily associated with cortical or deep grey matter injury (Himmelmann et al., 2017), which has a more significant impact on intellectual functioning than earlier insults (Cummins et al., 2021). This distinction underscores the complexity of CP and the multifactorial nature of its comorbidities (Vandenbosche & Kirchner, 1998). Furthermore, a less mature fetal brain may be better able to recover from early damage, compared to a term infant's brain (Hemming et al., 2008; O'Shea, 2008).

The severity of motor impairment, as measured by GMFCS levels, showed a strong correlation with the likelihood of ID (GMFCS III versus GMFCS I-II: OR = 2.2883; GMFCS IV-V versus GMFCS I-II: OR = 6.377), consistent with previous research (Bertoncelli et al., 2019; Reid et al., 2018). This highlights the importance of GMFCS as a critical tool not only for assessing motor function (Palisano et al., 1997), but also for predicting intellectual outcomes (Bertoncelli et al., 2019). A study recruited 148 children aged 5–10 years with IQ ranging from 50 to 69 and 300 children without intellectual limitations aged 5–10 years to explore predictive items from 75 milestones of the Dutch Developmental Instrument (Vlasblom et al., 2019). This study identified 10 milestones that could predict intelligence function, further demonstrating that motor function can influence cognition.

Regarding CP subtypes, mixed, quadriplegia, diplegia and dyskinesia all had higher probability of comorbidities with ID than hemiplegia, and the mixed had the highest probability (OR = 4.7096). A previous study reported that bilateral spastic CP (including diplegia and quadriplegia) had a 3.23 times higher likelihood of comorbidities ID than unilateral spastic CP (Cummins et al., 2021). A cross-sectional, observational study based on dyskinesia CP reported that 47.7% of children with dyskinesia CP had global delay (Saini et al., 2021), and a review reported that 50% to 60% of children with dyskinesia CP have an IQ less than 70(Stadskleiv, 2020).

Similarly, the presence of epilepsy were associated with higher risk of ID (OR: 1.8524, p<0.001), consistent with previous predictive models for severe ID in teenagers with CP (Bertoncelli et al., 2019). In the prediction model for epilepsy in CP children, a relevant association (OR: 2.698, p = 0.006) between epilepsy and profound ID was found (Bertoncelli et al., 2022).

Except for preterm birth, other predictors selected were all clinical features (GMFCS, MRICS and CP subtypes) or comorbidity (epilepsy). Etiology and high risks were not considered as important predictor for ID in CP children, similar to previous study (Levy-Zaks et al., 2014), underscoring the need to consider these clinical features in the management and prognosis of CP (Aguayo et al., 2019; Cummins et al., 2021; Olusanya et al., 2022).

A major strength of this study is the large sample size, which enhances the reliability and generalizability of the findings. Additionally, the use of rigorous statistical methods, including LASSO regression and bootstrapping validation, ensures the robustness of the model (Collins et al., 2015; van Straalen et al., 2021). The comprehensive inclusion of various potential risk factors—from maternal and prenatal factors to clinical features and comorbidities-adds depth to the predictive power of the model. However, the study has limitations. Firstly, this study is based on hospital data, which may not be entirely representative of the general population; Secondly, children with hypotonia subtype CP were excluded from our study based on the recruit criteria, which might affect the observed prevalence of ID in our CP cohort; Thirdly, we didn't conduct external validation. Additionally, some data were obtained from parental reports, which may introduce recall bias. Future study should consider a population-based approach to further validate the findings.

The study underscores the need for routine follow-up and early intervention in children identified at high risk of ID. Future research should aim to refine the predictive model further and explore the underlying mechanisms linking these predictors to ID. Additionally, expanding the study to diverse populations can enhance the model's applicability and effectiveness. Investigating the genetic and metabolic factors associated with term births leading to CP could provide deeper insights into the etiology of ID in these cases (Bertoncelli et al., 2019; Huang et al., 2016; Wang et al., 2024).

## CRediT authorship contribution statement

**Junying Yuan:** Conceptualization, Data curation, Investigation, Formal analysis, Investigation, Methodology, Writing – original draft. **Gailing Wang:** Data curation, Investigation. **Mengyue Li:** Data curation, Validation, Investigation. **Lingling Zhang:** Conceptualization. **Longyuan He:** Data curation, Validation, Investigation. **Yiran Xu:** Conceptualization, Resources, Data curation, Investigation, Validation, Supervision. **Dengna Zhu:** Data curation, Validation, Investigation, Resources, Data curation, Investigation, Validation, Supervision. **Zhen Yang:** Data curation, Validation, Investigation. **Wending Xin:** Data curation, Validation, Investigation. **Erliang Sun:** Data curation, Validation, Investigation. **Wei Zhang:** Data curation, Validation, Investigation. **Li Li:** Data curation, Validation, Investigation. **Xiaoli Zhang:** Data curation, Validation, Investigation. **Changlian Zhu:** Conceptualization, Writing – review & editing, Funding acquisition.

## Declaration of competing interest

The authors declare that they have no known competing financial interests or personal relationships that could have appeared to influence the work reported in this paper.

## References

[bib0001] Aguayo V., Verdugo M.A., Arias V.B., Guillen V.M., Amor A.M. (2019). Assessing support needs in children with intellectual disability and motor impairments: measurement invariance and group differences. Journal of Intellectual Disability Research : JIDR.

[bib0002] Attali E., Yogev Y. (2021). The impact of advanced maternal age on pregnancy outcome. Best practice & research. Clinical obstetrics & gynaecology.

[bib0003] Back S.A. (2017). White matter injury in the preterm infant: pathology and mechanisms. Acta neuropathologica.

[bib0004] Başaran A., Kilinç Z., Sari H., Gündüz E. (2023). Etiological risk factors in children with cerebral palsy. Medicine.

[bib0005] Bertoncelli C.M., Altamura P., Vieira E.R., Bertoncelli D., Thummler S., Solla F. (2019). Identifying factors associated with severe intellectual disabilities in teenagers with cerebral palsy using a predictive learning model. Journal of child neurology.

[bib0006] Bertoncelli C.M., Dehan N., Bertoncelli D., Bagui S., Bagui S.C., Costantini S., Solla F. (2022). Prediction model for identifying factors associated with epilepsy in children with cerebral palsy. Children (Basel, Switzerland).

[bib0007] Bufteac Gincota E., Jahnsen R., Spinei L., Andersen G.L. (2021). Risk factors for cerebral palsy in Moldova. Medicina (Kaunas, Lithuania).

[bib0008] Casey B.J., Tottenham N., Liston C., Durston S. (2005). Imaging the developing brain: what have we learned about cognitive development?. Trends in cognitive sciences.

[bib0009] Collins G.S., Reitsma J.B., Altman D.G., Moons K.G. (2015). Transparent reporting of a multivariable prediction model for individual prognosis or diagnosis (TRIPOD): the TRIPOD statement. BMJ (Clinical research ed.).

[bib0010] Cummins D., Kerr C., McConnell K., Perra O. (2021). Risk factors for intellectual disability in children with spastic cerebral palsy. Archives of disease in childhood.

[bib0011] Dalvand H., Dehghan L., Hadian M.R., Feizy A., Hosseini S.A. (2012). Relationship between gross motor and intellectual function in children with cerebral palsy: a cross-sectional study. Archives of physical medicine and rehabilitation.

[bib0012] Dolk H., Parkes J., Hill N. (2006). Trends in the prevalence of cerebral palsy in Northern Ireland, 1981-1997. Developmental medicine and child neurology.

[bib0013] Gong C., Liu X., Fang L., Liu A., Lian B., Qi X., Zhou S. (2023). Prevalence of cerebral palsy comorbidities in China: a systematic review and meta-analysis. Frontiers in neurology.

[bib0014] Hemming K., Colver A., Hutton J.L., Kurinczuk J.J., Pharoah P.O. (2008). The influence of gestational age on severity of impairment in spastic cerebral palsy. The Journal of pediatrics.

[bib0015] Himmelmann K., Beckung E., Hagberg G., Uvebrant P. (2006). Gross and fine motor function and accompanying impairments in cerebral palsy. Developmental medicine and child neurology.

[bib0016] Himmelmann K., Horber V., De La Cruz J., Horridge K., Mejaski-Bosnjak V., Hollody K., Krägeloh-Mann I. (2017). MRI classification system (MRICS) for children with cerebral palsy: development, reliability, and recommendations. Developmental medicine and child neurology.

[bib0017] Huang J., Zhu T., Qu Y., Mu D. (2016). Prenatal, perinatal and neonatal risk factors for intellectual disability: a systemic review and meta-analysis. PloS one.

[bib0018] Iasonos A., Schrag D., Raj G.V., Panageas K.S. (2008). How to build and interpret a nomogram for cancer prognosis. Journal of Clinical Oncology.

[bib0019] Lear B.A., Lear C.A., Dhillon S.K., Davidson J.O., Bennet L., Gunn A.J. (2022). Is late prevention of cerebral palsy in extremely preterm infants plausible?. Developmental neuroscience.

[bib0020] Lele S.R., Keim J.L. (2006). Weighted distributions and estimation of resource selection probability functions. Ecology.

[bib0021] Levy-Zaks A., Pollak Y., Ben-Pazi H. (2014). Cerebral palsy risk factors and their impact on psychopathology. Neurological research.

[bib0022] Li h., Rong Z., Shulian Z., Wenjing S., Weili Y., Xiaoli W., Quanfang Q. (2015). Chinese neonatal birth weight curve for different gestational age. Chinese Journal of Pediatrics.

[bib0023] Noten S., Rodby-Bousquet E., Limsakul C., Tipchatyotin S., Visser F., Grootoonk A., Roebroeck M.E. (2022). An international clinical perspective on functioning and disability in adults with cerebral palsy. Disability and health journal.

[bib0024] Novak I., Hines M., Goldsmith S., Barclay R. (2012). Clinical prognostic messages from a systematic review on cerebral palsy. Pediatrics.

[bib0025] O'Shea T.M. (2008). Diagnosis, treatment, and prevention of cerebral palsy. Clinical obstetrics and gynecology.

[bib0026] Olusanya B.O., Gladstone M., Wright S.M., Hadders-Algra M., Boo N.Y., Nair M.K.C., Davis A.C. (2022). Cerebral palsy and developmental intellectual disability in children younger than 5 years: Findings from the GBD-WHO Rehabilitation Database 2019. Frontiers in public health.

[bib0027] Palisano R., Rosenbaum P., Walter S., Russell D., Wood E., Galuppi B. (1997). Development and reliability of a system to classify gross motor function in children with cerebral palsy. Developmental medicine and child neurology.

[bib0028] Qian Z., Li T., Zhang Y., Chen S., Zhang H., Mickael H.K., Huang F. (2023). Prevalence of hepatitis E virus and its association with adverse pregnancy outcomes in pregnant women in China. Journal of Clinical Virology.

[bib0029] Reid S.M., Meehan E.M., Arnup S.J., Reddihough D.S. (2018). Intellectual disability in cerebral palsy: a population-based retrospective study. Developmental medicine and child neurology.

[bib0030] Riley R.D., Ensor J., Snell K.I.E., Harrell F.E., Martin G.P., Reitsma J.B., van Smeden M. (2020). Calculating the sample size required for developing a clinical prediction model. BMJ (Clinical research ed.).

[bib0031] Rosenbaum P., Paneth N., Leviton A., Goldstein M., Bax M., Damiano D., Jacobsson B. (2007). A report: the definition and classification of cerebral palsy April 2006. Developmental medicine and child neurology. Supplement.

[bib0032] Sadowska M., Sarecka-Hujar B., Kopyta I. (2020). Cerebral palsy: current opinions on definition, epidemiology, risk factors, classification and treatment options. Neuropsychiatric disease and treatment.

[bib0033] Saini A.G., Sankhyan N., Malhi P., Ahuja C., Khandelwal N., Singhi P. (2021). Hyperbilirubinemia and asphyxia in children with dyskinetic cerebral palsy. Pediatric neurology.

[bib0034] Sattoe J.N.T., Hilberink S.R. (2023). Impairments and comorbidities in adults with cerebral palsy and spina bifida: a meta-analysis. Frontiers in neurology.

[bib0035] Schneider R.E., Ng P., Zhang X., Andersen J., Buckley D., Fehlings D., Oskoui M. (2018). The association between maternal age and cerebral palsy risk factors. Pediatric Neurology.

[bib0036] Sigurdardottir S., Eiriksdottir A., Gunnarsdottir E., Meintema M., Arnadottir U., Vik T. (2008). Cognitive profile in young Icelandic children with cerebral palsy. Developmental medicine and child neurology.

[bib0037] Stadskleiv K. (2020). Cognitive functioning in children with cerebral palsy. Developmental medicine and child neurology.

[bib0038] Stoltzfus J.C. (2011). Logistic regression: a brief primer. Academic emergency medicine : official journal of the Society for Academic Emergency Medicine.

[bib0039] van Straalen J.W., Giancane G., Amazrhar Y., Tzaribachev N., Lazar C., Uziel Y., Swart J.F. (2021). A clinical prediction model for estimating the risk of developing uveitis in patients with juvenile idiopathic arthritis. Rheumatology (Oxford, England).

[bib0040] Vandenbosche R.C., Kirchner J.T. (1998). Intrauterine growth retardation. American family physician.

[bib0041] Vlasblom E., Boere-Boonekamp M.M., Hafkamp-de Groen E., Dusseldorp E., van Dommelen P., Verkerk P.H. (2019). Predictive validity of developmental milestones for detecting limited intellectual functioning. PloS one.

[bib0042] Wang Y., Xu Y., Zhou C., Cheng Y., Qiao N., Shang Q., Xing Q. (2024). Exome sequencing reveals genetic heterogeneity and clinically actionable findings in children with cerebral palsy. Nature medicine.

[bib0043] Willems M., van Berlaer G., Maes L., Leyssens L., Koehler B., Marks L. (2022). Outcome of 10 years of ear and hearing screening in people with intellectual disability in Europe: A multicentre study. Journal of applied research in intellectual disabilities : JARID.

[bib0044] Wolff R.F., Moons K.G.M., Riley R.D., Whiting P.F., Westwood M., Collins G.S., Mallett S. (2019). PROBAST: A Tool to Assess the Risk of Bias and Applicability of Prediction Model Studies. Annals of internal medicine.

[bib0045] Yuan J., Cui M., Liang Q., Zhu D., Liu J., Hu J., Zhu C. (2024). Cerebral palsy heterogeneity: clinical characteristics and diagnostic significance from a large sample analysis. Neuroepidemiology.

[bib0046] Yuan J., Song J., Zhu D., Sun E., Xia L., Zhang X., Zhu C. (2018). Lithium treatment is safe in children with intellectual disability. Frontiers in molecular neuroscience.

[bib0047] Yuan J., Wang J., Ma J., Zhu D., Zhang Z., Li J. (2019). Paediatric cerebral palsy prevalence and high-risk factors in Henan province, Central China. Journal of rehabilitation medicine.

